# Effects of Immigrants, Health, and Ageing on Economic Growth in the European Union

**DOI:** 10.3390/ijerph20010224

**Published:** 2022-12-23

**Authors:** Manuela Ortega-Gil, Chaima ElHichou-Ahmed, Antonio Mata-García

**Affiliations:** Department of General Economy, University of Cadiz, 11202 Algeciras, Spain

**Keywords:** immigrants, expenditure on health, subjective health, economic growth, ageing

## Abstract

Population ageing and low birth rates are two problems of the EU that have an impact on employment, production, and economic growth. Against this background, immigration, health expenditure, and the health of migrants must become a key element of European policy. For this reason, this paper focused on identifying the effect of immigration, health, and ageing on economic growth in order to highlight their importance from an economic perspective. We constructed different econometric models with Eurostat data for 27 countries and 13 years (2008–2020), whose dependent variable was gross domestic product. Independent variables were the number of immigrants by gender and age, health expenditure per capita (total and by function), immigrants’ perception of their health as very good, and the proportion of the population aged 65 years and over. The model selected to analyze the results was Prais–Winsten regression heteroskedastic panels corrected standard errors modeled by applying the option (ar1) to correct for autocorrelation, using Stata software (version 16). The results show that health expenditure has a significant positive effect on economic growth, higher in hospital services, followed by medical products; immigrants’ perception as very good is only significant in some models. The number of immigrants has a (positive) effect, although less significant than public expenditure on health. Its effect is larger when the immigrant is aged between 15 and 64 years and smaller for male immigrants than for female immigrants. Without the ageing variable, immigration is not significant. Moreover, there are significant differences between European countries in relation to the variables analyses (immigration, immigrants’ perception of their health, ageing and public expenditure on health, and public expenditure on health according to function). This may be due to the different regulations on both immigration and public health in the countries that make up the European Union.

## 1. Introduction

The European population is ageing rapidly. In 2011, the average age in the EU was 42 years and the percentage of people between 65 and 79 years was 12.9%; in 2021, the average age was 44 years and the percentage of people between 65 and 79 years was 14.8% with an upward trend. This trend is present in all the European countries analyzed. Therefore, the countries that make up the EU need immigrants to work and help economic growth. In addition, this ageing will cause an increase in healthcare costs and a healthy life must be promoted, so countries must be prepared to support this increase in healthcare expenditure. Eurostat defines immigration as the action by which a person establishes his or her usual residence in the territory of an EU country for a period that is at least 12 months, having previously been usually residing in another country.

The development of immigration policy in the EU is complex as it is based on directives and measures that have been changing to adapt to different situations. Articles 79 and 80 of the Treaty on the Functioning of the European Union (TFEU) provide the legal basis for immigration policy with the aim of creating a comprehensive European immigration policy based on solidarity, a forward-looking approach, and a balanced approach to both legal and irregular immigration. However, based on its competences, the EU can only encourage and support action by the Member States to promote integration between nationals and immigrants. The main reviews of European policy since 2008 focus on legal immigration, integration, and irregular immigration [1].

Immigrants have a positive impact on real Gross Domestic Product per capita (GDPpc) and the diversity effect seems to be more consistent in developing countries [2]. The study by Noja et al. (2018) [3] that used spatial analysis autoregressive models showed that flows of labor immigrants have important positive economic consequences, which involve changes in the labor market, in the GDPpc, and in the levels of employment and wages for both the native population and the foreign population, among other aspects. In contrast, the results of Boubtane et al. (2013) [4] showed that only in four countries (France, Iceland, Norway, and the United Kingdom) does economic growth have a positive effect on immigration, while in the rest of the countries analyzed immigration does not cause economic growth.

Irregular immigration depends on the legislation established by the countries, and the entry, stay, and the different situations experienced by migrants will depend on this legislation [5]. The management of irregular immigration is a priority both at national and European level, and in particular in those territories that perform the function of external borders of the European Union, such as Greece and Italy. Although fundamental rights must be respected, sometimes entry is restricted, as well as preventing people in need from applying for international protection [6], sometimes resulting in violations of the fundamental rights of asylum seekers at the external borders of the EU [7].

The legislation of Spain and Italy establishes that immigrants are welcomed with worker status, this causes their situation to depend on temporary work permits, which hinder a permanent stay while, at the same time, these countries carry out integration policies [8]. There is also no unified policy on the immigration of entrepreneurs, each country has different legislation on the renewal period, on the requirements for entrepreneurship, and on the period for making an investment [9]. European laws regarding immigration tend to be prohibitionist and punitive [10]. In addition, the attitudes of national citizens towards immigrants vary across regions. Those with the most positive attitudes are some of the Spanish, French, and German regions [11]. Ledoux et al. (2018) [12] studied the inclusive policies in health for migrants in Ireland, Spain, and Portugal.

Cooperation with non-EU countries is one of the ways to manage migration flows as they appear in the existing regulations [13]. The EU makes cooperation agreements, such as the Migration Partnership Framework to reduce irregular migration and encourage the return of undocumented migrants. In the case of West Africa, the countries that constitute the EU try to satisfy their own interests through international funding, while complying with the agreements reached. These countries oppose forced returns, while in other territories they adapt and reinterpret their initial political interests or develop other measures such as the implementation of improvements in border control [14]. Migrants arriving in receiving territories face various physical and socioeconomic situations that affect their health [15]. Therefore, as stated by Puchner et al. (2018) [16], inclusive policies with a sustainable and comprehensive health approach are needed in Europe.

In the coming years, the increase in the number of migrants may be associated with increased health problems in the population [17]. Migrants experience vulnerabilities that have counterproductive effects on their health, such as fear, chronic anxiety, low self-esteem, and loss of control, among others [18]. “Although the overall number of HIV diagnoses in migrants from high-prevalence countries have declined in the EU/EEA over the past decade, migrants still accounted for 40% of the reported cases in 2016 (range 1–80%)” [19] (p. 2). In this situation, rapid testing realizations can help in the improvement of public health, early diagnosis of the disease, as well as the implementation of treatments that improve health and reduce mortality.

In Italy, migrants who are engaged in agriculture and live in settlements face a lack of housing and sanitation and suffer segregation and racism. This situation prevents them from accessing comprehensive healthcare in Italy [20]. Another study shows that in Greece, despite European funding, psychological services and other health issues are deficient while Italy performs better in terms of the quality of health service provision. The increased mental and psychological stress of migrants in reception centers makes these services necessary [21].

Another study comparing Germany and the United States shows a negative impact on the subjective mental and physical health of undocumented immigrants due to their legal status causing stress and exclusion from health services. Furthermore, it indicates that in many countries, access to public health services and stress problems due to fear of deportation can aggravate physical and mental illnesses. These result in high morbidity and mortality rates in the immigrant population, although there are differences according to region of origin, educational level, gender, and migration experience [22]. The study by La Parra-Casado (2017) [23] on the subjective health of immigrants indicates that both men and women consider their health to be good. As for second-generation, only men had a greater deterioration in perceived health by age, while the perceived health of older immigrants was similar to natives.

Authors such as Bloom et al. (2018) [24] have argued that less developed and post-demographic transition countries are those where health has the strongest positive effect on economic growth, particularly the health of children and women, and actions to improve the health of women and children can have significant benefits in terms of economic growth, well-being, and long-term development. Studies have shown that states can improve people’s health through subjective well-being [25]. Such policies are necessary, as immigrants have more physical ailments and higher levels of anxiety and depression, and their self-perceived health is lower than that of natives [26,27].

The European Union has shared competences in the field of health with its Member States. Article 168.7 of the TFEU states that EU Member States are responsible for the allocation of resources for the management of health services and medical care. Public health in the EU represents a system that guarantees curative and preventive health [28]. The Classification of the Functions of Government (COFOG) divides public expenditure on health into expenditures on health medical products, health outpatient services, health hospital services, health services, public health services, and basic research and development and administration. The study by Serban, Stoenoiu, and Cristea (2020) [29] highlighted the importance of public expenditure on health in terms of its size and necessity, especially hospital services and outpatient services. Despite its importance, public expenditure on health in the EU-27 decreased in the period 2009–2019 from 7.3% of GDP to 7.0% [30].

Authors Lupu et al. (2018) [31] and Kutasi and Marton (2020) [32] showed a positive relationship in their studies between public expenditure on health and economic growth. Kutasi and Marton (2020) [32] analyzed the effect of functional public expenditure (COFOG) using different methods (general moment difference, panel fixed effects model and ordinary least squares model) with the result of a positive relationship between both variables. In contrast, Bania et al. (2007) [33] and Boldeanu and Ianu (2016) [34] showed an inverse relationship between public expenditure on health and economic growth. Along the same lines are the studies of Boldeanu et al. (2015) [35] that presented negative results in the study of 30 European countries and Sengupta (2022) [36] which, through a dynamic estimation analysis, shows that public expenditure on health negatively affects growth in developed economies and positively in less developed economies, in the long run. In the short run, causal relationships are not possible in both cases. Other research reports that the sign depends on different structural factors [37,38].

The relationship between private health expenditure and economic growth has also been studied. The study by Halıcı-Tülüce et al. (2016) [39] indicated an inverse relationship between private health expenditure and economic growth and raised the need to implement a decision system that links expenditure and income decisions and ensures the use of resources in a transparent manner. Holmberg and Rothstein (2011) [40] presented a slightly negative relationship between private health expenditure and good health of the population and stressed the need to pay attention to the public health sector based on the allocation of funds for public health employment.

According to Serban et al. (2020) [29], public policies must take into account migration, demographic changes, population ageing, and the structure of public expenditure, among others. This paper focuses on these phenomena in the 28 countries that make up the European Union in the period 2008–2017 in relation to economic growth. The literature review detects studies with opposing results, some authors presenting a positive relationship between immigration and economic growth and others the opposite situation due to public expenditure on health. For this reason, we believe it is necessary to present these three problems facing the European Union, immigration, the ageing of the population, and health in relation to economic growth. 

Consequently, the aim of this paper is to identify the effects of immigration, the ageing of the European population, and health on economic growth. For this purpose, the dependent variable is GDPpc, and immigration, health, and ageing are the independent variables. Four variables represent immigration (the number of immigrants, as well as their breakdown by gender and age between 15 and 64, as this is the working age according to Eurostat). Health is analyzed from two perspectives: public expenditure on health according to the Functional Classification of Public Administration Expenditure (COFOG) and the perception of health as very good by immigrants. Likewise, the variable representing ageing is the proportion of population aged 65 years and over of the EU member countries. By reviewing the current state of the literature on the economic consequences of immigration, health, and ageing, in compliance with our general research objective, we defined the following research hypotheses:

**Hypothesis 1** **(H1).** *There is a direct positive relationship between the number of migrants and economic growth (defined through the GDP per capita) by country in the EU, higher for women immigrants than for men immigrants*.

**Hypothesis 2** **(H2).** There is a positive direct relationship between working-age immigrants and economic growth (defined through the GDP per capita) by country in the EU.

**Hypothesis 3** **(H3).** *Public expenditure on health has positive effects on economic growth (defined through the GDP per capita) by countries in the UE. Higher in hospital and outpatient services*.

**Hypothesis 4** **(H4).** *Ageing (the proportion of population aged 65 years and over) has significant positive effects on economic growth (defined through the GDP per capita) by country in the EU*.

## 2. Materials and Methods

Data are from the Eurostat database, with 351 observations, 27 European Union countries and 13 years (2008–2020). The countries included are Austria, Belgium, Bulgaria, Croatia, Cyprus, Czechia, Denmark, Estonia, Finland, France, Germany, Greece, Hungary, Ireland, Italy, Latvia, Lithuania, Luxembourg, Malta, Netherlands, Poland, Portugal, Romania, Slovakia, Slovenia, Spain, and Sweden.

The variables selected are Gross Domestic Product per capita (GDPpc), the number of immigrants, health perceived as very good represented, public expenditure on health per capita, and percentage of population aged 65 years or older over total population. These data create a panel matrix containing 27 countries, 13 years, and the variables selected for the econometric analysis. Immigration variables are four of the total number of immigrants, the first is total immigrant (Immigrants), the second women immigrants (Immigrantswom), the third men immigrants (Immigrantsmen) and the fourth the number of immigrants between 15 and 64 years of age (Immigyears15_64). Representing health used public health expenditure per capita (Healthpc) and its functional breakdowns (Health_Medical_productspc, Health Outpatient_servicespc, Health_Hospital_servicespc, Health_Public_health_servicespc, logHealth_R&D_Healthpc, Health_n.e.cpc) and health perceived as very good among migrants aged 16–64 years (HealthImmig_VGood16_64). The indicator to represent ageing is the proportion of the population aged 65 years and over of each country (Ageing_65). All datasets were introduced in the econometric software Stata (version 16) and several models were estimated. For better estimation, the variables GDPpc, the number of migrants, women migrants, men migrants and migrants aged 15–64 years, as well as public expenditure per capita on health and its functional breakdown were used logarithmically. We also created a time variable that divides the years into three periods. The first, economic crisis (2008–2012), the second, recovery (2013–2017), and the third of stagnation (2018–2020) pre-COVID-19. The description and characteristics of the variables selected for the models are in Table 1.

Models were estimated following the guidelines by [41,42,43,44,45,46,47], these models are represented [48,49,50,51,52,53]:

- One Model Linear regression. The form of the regression equation is shown in Equation (1) below.

(1) $$logGDPpc_{it}=\alpha \;+\beta_{1}logIMMIG_{it}+\beta_{2}logE.HEALTH_{it}+\beta_{3}S.IMMIG.HEALTH_{it}\;+\beta_{4}AGEING_{it}+\varepsilon_{it}$$

where *i* are the countries; *t* the years; *GDPpc* is the dependent variable of each country (*i*) each year (*t*); *β* are the estimators of the predictor variables immigrants (*IMMIG*), expenditure on health (*E.HEALTH*), subjective health of the immigrants (*S.IMMIG.HEALTH*), and the ageing (*AGEING*); *α* is the intercept or constant; and εit is the disturbance or error term.

- Two Model Panel, one Fixed Effects (FEs) and other Random Effects (REs). In FE the error (*εit*) can be decomposed into two parts: a fixed, constant part for each individual (*vi*) and a random part that meets the OLS requirements (*uit*). Therefore *εit* = *vi* + *uit*, which is equivalent to obtaining a general trend by regression giving each individual a different point of origin (ordinates). The Stata program calculates this by means of the difference, also decomposing the variance into two: intro and between groups. Into two: intro and between groups (Equation (2)). The random effects model (RE) has the same specification as the fixed effects model except that vi, instead of being a fixed value for each individual and constant over time for each individual, is a random variable with a mean value vi and a variance Var (vi) ≠ 0, vi is a random variable. This model is more efficient but less consistent than the fixed effects model.

(2) $$logGDPpc_{it}=\alpha +\beta_{1}logIMMIG_{it}+\beta_{2}logE.HEALTH_{it}+\beta_{3}S.IMMIG.HEALTH_{it}\;+\beta_{4}AGEING_{it}+\nu i+uit$$

- One Linear regression, absorbing indicators. This model fits a linear regression that absorbs a categorical factor. It is designed for data sets with many groups, but not a number of groups that increases with sample size. The form of the regression equation is shown in Equation (3) below. For instance, the dummy variables can indicate countries β the estimators of the predictor variables (Equation (3)).

(3) $$logGDPpc_{it}=\alpha +\beta_{1}logIMMIG_{it}+\beta_{2}logE.HEALTH_{it}+\beta_{3}S.IMMIG.HEALTH_{it}\;+\beta_{3}AGEING_{it}+d1\gamma 1+d2\gamma 2+\cdots +dk\gamma k+\varepsilon \;$$

- Prais–Winsten regression, heteroskedastic panels corrected standard errors. This model estimates the rest of the models.

From the comparison of alternative models, we may conclude that the most appropriate among the models considered is the Prais–Winsten estimation with ar1. In addition, for this reason this model was the starting framework for the rest models. Once the four previous models were made, tests of heteroskedasticity and autocorrelation in the models were carried out and these problems were presented. The Prais–Winsten regression model, and heteroskedastic panels corrected standard errors, was used to correct them. 

This method estimates seventeen models. Four capture the differences between the types of immigrants, namely model 5, immigrants in general; model 6, women immigrants; model 7, men immigrants; and model 8, immigrants aged between 15 and 64 years. Six models detect the effect of health expenditures according to their function models 9, 10, 11, 12, 13, and 14 representing expenditures on health medical products, health outpatient services, health hospital services, health services, public health services, and basic research and development and administration, respectively. Finally, we estimated seven models to introduce time and country variables into the Prais–Winsten regression model.

This model is an alternative to Generalized Least Squares for fitting linear cross-sectional time series models whose disturbances are heteroskedastic and correlated in all panels. The disturbances can also be assumed to be autocorrelated within panel, and the autocorrelation parameter can be constant across panels or different for each panel. This model can write Equation (4):

(4) $$y_{it}=\alpha +x_{it}\beta +\epsilon_{it}$$

This model assumes that the disturbances are, by default, heteroskedastic and contemporaneously correlated across panels. *i* = 1. *m* is the number of units (or panels); *t* = 1. *Ti*; *Ti* is the number of periods in panel *i*; and *εit* is a disturbance that may be autocorrelated along t or contemporaneously correlated across i. We use the option common correlation coefficient (ar1) to correct the autocorrelation and het to heteroskedastic. When autocorrelation with a common coefficient of correlation is specified (ar1) this is computed as Equation (5):

(5) $$\rho =\frac{\;\rho_{1}+\rho_{2}+\cdots +\rho_{m}}{m}\;$$

where *ρ_i_* is the estimated autocorrelation coefficient for panel, and *i* and *m* is the number of panels. The covariance of the OLS or Prais–Winsten coefficients is:

(6) $$Var\;\left(\beta \right)={\left(X^{\prime }X\right)}^{-1}\;X^{\prime }Ω\;X\;{\left(X^{\prime }X\right)}^{-1}$$

where Ω is the full covariance matrix of the disturbances. When the panels are balanced, we can write Ω as Equation (7). We used xtpcse and estimated the elements of Σ as Equation 8:

(7) $$Ω=\Sigma_{m\times m\;}⊗\;I_{T_{i}\times T_{i}}$$

(8) $$\hat{\sum_{ij}}=\frac{{\epsilon^{\prime }}_{i}\epsilon_{j}}{T_{ij}}$$

In the Equation (8), *i* and *j* are the residuals for panels *i* and *j*, respectively, that can be matched by period; and where *T_ij_* is the number of residuals between the panels *i* and *j* that can be matched by time period [49]. We also included (Equation (9)) dummy variables to control for country *i* and wave *t* fixed effects in vectors *I* (*COUNTRYj*) and *I* (*WAVEt*) and cluster standard errors at the country level (*ε_it_*).

(9) $$logGDPpc_{it}=\alpha \;+\beta_{1}logIMMIG_{it}+\beta_{2}logE.HEALTH_{it}+\beta_{3}S.IMMIG.HEALTH_{it}+\beta_{3}AGEING_{it}+I\left(COUNTRY_{j}\right)\beta_{h+1}+I\left(WAVE_{t}\right)\beta_{h+2}+\varepsilon_{it}\;$$

## 3. Analysis and Results

The dependent variable in all the models is GDPpc and the independent variables differ according to the models. In the first five models, the independent variables are the number of immigrants, public expenditure on health, the percentage of immigrants who perceive their health as very good, and the proportion of population aged 65 years and over. In models 6, 7, and 8 the immigration variable changes to immigrant women (model 6), immigrant men (model 7), and number of immigrants between 15 and 65 years of age (model 8). The other variables (public expenditure on health, the percentage of immigrants who perceive their health as very good, and the proportion of population aged 65 years and over) continue to be the same. This change in the independent variable immigration is to analyze the effect of gender and age of immigrants on economic growth.

In order to detect the effect of public expenditure on health according to its function, six models were created, one for each expenditure function, expenditure on health products (model 9), ambulatory health services (model 10), hospital health services (model 11), health services (model 12), public health services (model 13), and basic research and development and administration (model 14). In these models, the remaining independent variables are the number of immigrants, the perception of immigrants’ health as very good, and ageing. In addition, to capture the country and period effect, we created seven new models, one for each type of public expenditure. For expenditure on health (model 15), expenditure on health products (model 16), ambulatory health services (model 17), hospital health services (model 18), health services (model 19), public health services (model 20), and basic research, development, and administration (model 21). In addition, we estimated four models with each immigration variable without introducing the ageing variable in the models (models 22–25).

In summary, we developed 25 models. The first five to detect the most appropriate for the study (models 1–5), three to analyze the effect on economic growth of the gender and age of immigrants (models 6–8), six to analyze the effect on economic growth of expenditure on health according to its function (models 9–14), seven to analyze the significance according to the period and the 27 EU countries (models 15–21). We considered three periods, the first one representing the economic crisis (2008–2012), the second one of recovery (2013–2017), and the third one of stagnation (2018–2020). Finally, we estimated four models to determine the effect of migration on economic growth without the ageing variable (models 22–25).

The first estimation was performed using an OLS and the Skewness and kurtosis tests were performed for normality (Prob > chi2 = 0.000), Breusch–Pagan test for heteroscedasticity (Prob > chi2 = 0.7623), and the Ramsey test (Prob > F = 0.000). Akaike’s test AIC = −218.9635 BIC = −199.9228 and Variance Inflation Factor (VIF) = 1.28 and of all the independent variables the highest VIF is logImmigrants with 1.45 and since the results are close to 1, there is no correlation between the predictor variables, the variables are weakly correlated. 

Subsequently, the model variables were estimated using Random Effects (REs) and Fixed Effects (FEs) (models 2 and 3) and the Breusch–Pagan Lagrangian multiplier test for random effects was performed, with the result = 0.0000, which indicates that a panel model should be used. Subsequently, we applied the Hausman test with the result of chi2 < 0 (−2.70). Therefore, the model fitted on these data fails to meet the asymptotic assumptions of the Hausman. To solve this situation, we performed the testparm test with the result Prob > F = 0.0000 for all years, so we rejected the null that the coefficients for all years are jointly equal to zero, so time-fixed effects are needed. Therefore, to control for fixed effects we calculated the linear regression, absorbing (country) in model 4 with the same results as FE model. However, we also applied to the models the modified Wald test for heteroscedasticity by groups in the fixed effects regression model with the result of Prob > chi2 = 0.0000; therefore, the model presents heteroscedasticity and the Wooldridge test for autocorrelation in panel data whose result is Prob > F = 0.0000 showing the existence of autocorrelation.

Finally, to correct for heteroscedasticity and autocorrelation, we estimated the Prais–Winsten regression model, with standard errors corrected for heteroscedastic panels (models 5 and following). Once the most correct model was identified, we proceeded to run four models to identify the effect of immigration by gender and age (models 5–8). We ran six models to see the effect of different functional health expenditures (models 9–14). Next, the testparm was run for the time wave variables and the country variable with the result Prob > F = 0.0000 for all waves and countries. Therefore, to control and estimate the time and country effects, 7 more models were run, one for health expenditure and 6 for health expenditure by function (models 15–21). Table 2, Table 3, Table 4 and Table 5 show the results of all models.

From the results of the Prais–Winsten regression models, it is worth noting that the variable public expenditure on health presents a positive significance *p* < 0.01, being its effect on GDP of 0.716 *** without the time and country effect and 0.407 *** when these fixed effects are introduced. Therefore, a 1% increase in public expenditure on health is associated with a 0.407% change in GDP (Table 3 and Table 5). In their functional breakdown all models (Table 4 and Table 5) show a positive significance *p* < 0.01 with the largest effect of health expenditure on hospital services per capita, followed by medical products. Thus, controlling for fixed effects, a 1% increase in public health expenditure on hospital services per capita is associated with a 0.365% change in GDP and a 1% increase in public health expenditure on medical products per capita is associated with a 0.246% change in GDP (Table 5). On the other hand, the perception of health as very good among immigrants aged 16 to 64 years is not significant in almost all models.

With respect to immigrants in the EU, the analysis shows a positive significance at *p* < 0.05 for immigrant women and immigrants aged 15–64 years, and positive at *p* < 0.1 for immigrant men. The effect on economic growth was greater for immigrant women (0.0190 **) than for immigrant men (0.0169 *). The effect of immigrants aged 15–64 years (0.0318 **) is larger than gender on economic growth (models 6, 7, and 8). The effect of immigrants on GDP increases and is significant *p* < 0.01 in the models that have functionally disaggregated public expenditure on health and where period and country were considered as a control variable. The largest effect is found in model 12 (Table 4) with 0.0847 ***; therefore, a 1% increase in immigrants is associated with a 0.084% change in GDP (Table 4).

The analysis of the ageing factor shows relevant differences depending on whether the model contains fixed effects (period, country, Table 5) and public expenditure on health by functions (Table 4) or public expenditure on health (Table 3). In Table 5, which is broken down by functions with time and country control variables, the effect of the proportion of population aged 65 years and over on GDP is positive and significant *p* < 0.01, with the effect of ageing being larger in model 19 (0.0505 ***). This indicates that a one-unit increase in the percentage of people 65 years and over is associated with a 5.05% change in GDP. This model differs from the others in that it analyzes the effect on GDP by introducing the functional public expenditure “Public health services” and the control variables of time and country. However, in the models without public expenditure on health broken down by function (Table 3) the effect of ageing on GDP is significantly negative *p* < 0.01. It is also significantly negative at *p* < 0.01 in model 11 when in the same model as public expenditure on hospital services (Table 4). Another very important point is the effect of introducing the ageing variable on immigration; Table 6 shows that when the ageing variable is not introduced in the models, immigration in all the variants analyzed becomes non-significant for economic growth.

Table 5 introduces fixed effects of time (wave) and countries. The results show that there is a positive time effect. Mainly in period 3 (years 2018–2020) as it is significantly positive *p* < 0.01 in almost all models. Period 2 (years 2013–2017) is only significantly positive *p* < 0.05 in models 15 (includes public expenditure on health) and 18 (includes public expenditure on hospital services). There is also a country effect, as the countries are mostly significant *p* < 0.01 in all models in Table 5, the base country is Austria, and the countries with the highest positive effect of the analyzed variables are Luxembourg followed by Ireland, Cyprus, and Denmark. Luxembourg and Denmark are the two countries with the highest per capita public expenditure on health and Ireland and Cyprus are among the countries with the lowest percentage of elderly people and the highest percentage of immigrants in very good health. In the opposite direction, Bulgaria, Romania, Croatia, Poland, and Hungary have a greater negative effect. These countries are the ones that in the average of the coefficients of the models in Table 5 are the most with negatively distanced from the base country Austria (Table 7). Therefore, the variables of the models in Table 5 as a whole in these countries have a smaller effect on economic growth than the rest of the countries. Another noteworthy aspect is the reduction in the coefficient of public expenditure on healthcare for outpatient services (logHealth_Outpatient_servicespc) when the country and period effects are introduced, from 0.308 *** to 0.0518 **.

## 4. Discussion

The results confirm the hypotheses stated in the study. They confirm hypothesis 1 as it shows that there is a positive relationship between immigration and economic growth; these results are in line with the study conducted by [2]. However, they differ from those of [4], which indicates that immigration does not lead to economic growth. This may be due to the fact that in our study we included the variable ageing, without it the results would be different, immigration would not be significant for economic growth. In addition, we considered that it was necessary to include gender in immigration and it is relevant that the effect of immigrant women is higher than that of immigrant men. It would be interesting to delve further into the causes which lead to this situation. 

Another aspect to highlight is that the number of immigrants of working age (15–64 years) is the immigration variable that has the greatest positive effect on economic growth (therefore, hypothesis 2 is confirmed) and it is in line with the study by Noja et al. (2018) [3] which showed that flows of labor migrants have significant positive economic consequences. In this situation, a unified immigration and labor market policy would be advisable, as recommended by Calavita (2017) [8]. It would also be necessary that the requirements for an immigrant to be an entrepreneur be homogenous in the EU (De Lange, 2018) [9]. As indicated in this paper, the EU population is ageing and the labor market, both from the demand side (entrepreneurs) and the supply side (workers), need immigrants of working age who, among other things, contribute value to GDP.

The results also present a significant and positive relationship of public expenditure on health and economic growth, the analysis according to functional health expenditure shows that hospital services followed by health products have the largest positive effects on economic growth. These results confirm hypothesis 3 and are in line with studies conducted by Lupu et al. (2018) [31] and Kutasi and Marton (2020) [32]. Moreover, the study by Serban et al. (2020) [29] provided a positive relationship, although it differs from our work in the order of importance of health expenditures. In their case, outpatient services are in second place, while in ours, health products are in second place. In contrast, studies by Bania et al. (2007) [33], Boldeanu and Ianu (2016) [34], and Sengupta (2022) [36] demonstrate an inverse relationship between public health expenditure and economic growth. On the other hand, the perception of health as very good among immigrants aged 16–64 years is not significant in almost all models. These results are in line with the work of Wittig et al. (2008) [26] and Nielsen et al. (2010) [27], which indicates that immigrants have more physical ailments and higher levels of anxiety and depression, and their self-perceived health is lower than that of natives. The COVID-19 crisis has shown that health and economic growth are highly correlated. It would be interesting to incorporate a fourth period in the paper (2020–2022) that captures the effect of COVID-19 on public expenditure, health, and economic growth.

The ageing of the EU population is a reality as shown by Eurostat data. In our study, their results are different depending on the variables estimated in the model. When public expenditure on health is disaggregated by function and the control variables of time and country are introduced, its effect on economic growth is positive and significant, being greater when functional public expenditure on public health services is considered, although the expenditure is not significant. This model captures the difference between countries, hence the effect of ageing. These differences between the results of the models show that the effect of ageing is heterogeneous between the countries that make up the EU. As indicated above, the introduction of this variable in the models makes the immigration variables significant. Therefore, hypothesis 4 is confirmed when the time and country control variables are introduced.

Finally, the results by country show that Luxembourg and Denmark are the two countries with the highest public expenditure on health per capita, and Ireland and Cyprus are among the countries with the lowest percentage of elderly people. The highest percentage of immigrants in very good health are the countries with the highest positive effect on economic growth, while those with the lowest public expenditure (Bulgaria and Romania) are the countries with the lowest.

## 5. Conclusions

This paper provides information that can be useful in the elaboration of European policies oriented towards immigration, health, and ageing. The analysis shows that the effect of immigration on GDP in the European Union is positive and significant. It is higher when the immigrant is aged between 15 and 64 years and lower for men immigrants than for women immigrants. These data reflect the importance for economic growth of immigration in the EU, mainly when immigrants are of working age and higher for women than for men. The results also show that without the ageing factor, immigration is not significant for economic growth. Therefore, this study provides an objective economic perspective on immigration, which on the one hand, serves to justify the need for immigrants in the European economy in the face of anti-immigration discourses. On the other hand, it is useful for improving the economic efficiency of the immigration policy as it shows the need for a gender perspective and for enhancing the productive sector-oriented inflow of working-age immigrants in immigration policymaking. De Lange (2018) [9] in her study showed the barriers to work and entrepreneurship in the immigration regulations of European countries. Our study indicates that improving and facilitating work and entrepreneurship for 15–64-year-olds would lead to economic growth. Therefore, it would be interesting to analyze the effect on economic growth, well-being, and health of immigrants through economic activity and entrepreneurship.

Our results also reflect the positive effect of public health spending on economic growth, especially spending on hospital services and medical products. This shows that the higher the public health expenditure, the higher the economic growth. With the introduction of the country and time factor, the coefficient and significance of public health expenditure on outpatient services decreases considerably.

Furthermore, the study presents the existence of significant differences between European countries in relation to the variables analyzed (immigration, perception of health with immigrants, ageing, and public expenditure on health, and public expenditure on health according to their function). This may be due to the different regulations on both immigration and public health in the countries that make up the European Union. Consequently, a homogenization of these policies among EU member states would be advisable.

To conclude, in this paper we demonstrate that the proportion of the population aged 65 years and over is expected to increase in the EU and the effectiveness and efficiency of public expenditure on health is being questioned. This study shows its positive effect on economic growth and the need to increase expenditure on health. It is remarkable that the introduction of the ageing variable in the model causes the immigration variables to be significant in relation to economic growth, which indicates an interrelation between both variables and highlights the need to improve immigration policies and to study whether public expenditure on health can be considered as an investment rather than an expense.

## Figures and Tables

**Table 1:** Description of variables.

Dependent Variables (Observations)	Description	Mean (Std. Dev)	Min (Max)
logGDPpc (351)	Logarithm that represents the dependent variable, the Gross Domestic Product per capita.	9.997754 (0.6439774)	8.492901 (11.53908)
**Independent** **Variables** **(Observations)**	**Description**	**Mean** **(Std. Dev)**	**Min** **(Max)**
logImmigrants (345)	Logarithm of number of immigrants. Name data Immigration by age and gender code: MIGR_IMM8.	10.88242 (1.351129)	7.878155 (14.26725)
logImmigrantswom (345)	Logarithm of number of women immigrants. Name data in Immigration by age and gender code: MIGR_IMM8.	10.27729 (1.332269)	7.271704 (13.80309)
logImmigrantsmen (345)	Logarithm of number of men immigrants. Name data in Immigration by age and gender code: MIGR_IMM8.	10.07519 (1.391784)	7.06732 (13.27661)
logInmigyears15_64 (191)	Logarithm of number of immigrants, from 15 to 64 years. Name data in Immigration by age and gender code: MIGR_IMM8.	10.71917 1.21558	8.110427 13.31669
logHealthpc (351)	Logarithm of expenditure on health per capita.	7.194276 (0.7982073)	5.289693 (8.702002)
PHealthInmig_VGood16_64 (339)	The self-perceived health of foreign country from 16 to 64 years as very good (percentage) code: HLTH_SILC_23.	25.64071 (14.75465)	1 (70.4)
logHealth_Medical_productspc (351)	Logarithm of expenditure on covers medicaments, prostheses, medical appliances, and equipment and other health-related products obtained by individuals or households, either with or without a prescription, usually from dispensing chemists, pharmacists, or medical equipment suppliers.	5.220587 (1.018012)	1.488151 (7.59705)
logHealth_Outpatient_servicespc (351)	Logarithm of expenditure on expenditure on covers medical, dental, and paramedical services delivered to outpatients by medical, dental, and paramedical practitioners and auxiliaries. The services may be delivered at home, in individual or group consulting facilities, dispensaries or the outpatient clinics of hospitals and the like.	5.642796 (1.270702)	1.669467 (7.398692)
logHealth_Hospital_servicespc (351)	Logarithm of expenditure on hospitalization is defined as occurring when a patient is accommodated in a hospital for the duration of the treatment. Hospital day-care and home-based hospital treatment are included, as are hospices for terminally ill persons.	6.49218 (0.738873)	4.631555 (8.116989)
logHealth_Public_health_servicespc (344)	Logarithm of expenditure on public health services.	3.078756 (1.411965)	−2.154098 (5.908472)
logHealth_R&D_Healthpc (324)	Logarithm of expenditure on basic research, applied research and experimental development.	2.482447 (1.997765)	−3.054239 (5.586825)
logHealth_n.e.cpc (351)	Logarithm of expenditure on administration, operation, or support of activities such as formulation, administration, coordination, and monitoring of overall health policies, plans, programmers, and budgets. Preparation and enforcement of legislation and standards for the provision of health services, including the licensing of medical establishments and medical and paramedical personnel; production and dissemination of general information, technical documentation, and statistics on health.	3.6126 (1.121843)	−1.409938 (6.106812)
Ageing_65 (351)	The proportion of population aged 65 years and over.	17.76917 2.485725	10.77643 23.23733
Wave (351)	A time variable that divides the years into three periods. The first, economic crisis (2008–2012); the second, recovery (2013–2017); and the third, stagnation (2018–2020) pre-COVID-19.	1.846154 (0.7703289)	1 3
Country (351)	This is the variable representing the 27 EU countries. These are: Austria, Belgium, Bulgaria, Croatia, Cyprus, Czechia, Denmark, Estonia, Finland, France, Germany, Greece, Hungary, Ireland, Italy, Latvia, Lithuania, Luxembourg, Malta, Netherlands, Poland, Portugal, Romania, Slovakia, Slovenia, Spain, and Sweden.		

Own elaboration by the authors.

**Table 2:** OLS, RE, FE, and OLS Absorb (country) all robust models.

Models	OLS (Robust)	RE (Robust)	FE (Robust)	OLS Absorb (Country)
	Model 1	Model 2	Model 3	Model 4
Variables	logGDPpc	logGDPpc	logGDPpc	logGDPpc
logImmigrants	−0.0174 *** (0.00644)	0.0439 *** (0.0154)	0.0633 *** (0.0207)	0.0633 *** (0.0120)
logHealthpc	0.772 *** (0.0158)	0.603 *** (0.0710)	0.476 *** (0.0841)	0.476 *** (0.0326)
PHealthInmig_VGood16_64	0.00420 *** (0.000901)	0.00114 (0.00130)	0.000586 (0.00117)	0.000586 (0.000855)
ageing_65	−0.0152 *** (0.00458)	0.0211 *** (0.00638)	0.0305 *** (0.00847)	0.0305 *** (0.00384)
Constant	4.793 *** (0.120)	4.778 *** (0.388)	5.348 *** (0.438)	5.348 *** (0.188)
Observations	333	333	333	333
R-squared	0.922		0.772	0.989
Number of Idcountry		27	27	

Own elaboration by the authors using Stata. Standard errors in parentheses *** *p* < 0.01.

**Table 3:** Prais–Winsten Regression models with different variables representing immigration.

Models	Prais–Winsten Regression	Prais–Winsten Regression	Prais–Winsten Regression	Prais–Winsten Regression
	Model 5	Model 6	Model 7	Model 8
Variables	logGDPpc	logGDPpc	logGDPpc	logGDPpc
logImmigrants	0.0187 **			
	(0.00910)			
logImmigrantswom		0.0190 ** (0.00925)		
logImmigrantsmen			0.0169 * (0.00922)	
logImmigyears15_64				0.0318 **
				(0.0128)
logHealthpc	0.716 *** (0.0216)	0.715 *** (0.0219)	0.717 *** (0.0214)	0.815 *** (0.0241)
PHealthImmig_VGood16_64	0.000907 (0.000831)	0.000871 (0.000829)	0.000937 (0.000833)	−0.000675 (0.000831)
Ageing_65	−0.0226 *** (0.00646)	−0.0225 *** (0.00647)	−0.0225 *** (0.00646)	−0.0288 *** (0.00671)
Constant	5.013 *** (0.172)	5.033 *** (0.169)	5.028 *** (0.172)	4.315 *** (0.201)
Observations	333	333	333	179
R-squared	0.996	0.996	0.996	0.998
Number of Idcountry	27	27	27	

Own elaboration by the authors using Stata. Standard errors in parentheses *** *p* < 0.01, ** *p* < 0.05, * *p* < 0.1.

**Table 4:** Prais–Winsten Regression models with breakdown of public health expenditure by function.

Models	Prais–Winsten Regression	Prais–Winsten Regression	Prais–Winsten Regression	Prais–Winsten Regression	Prais–Winsten Regression	Prais–Winsten Regression
	Model 9	Model 10	Model 11	Model 12	Model 13	Model 14
Variables	logGDPpc	logGDPpc	logGDPpc	logGDPpc	logGDPpc	logGDPpc
logImmigrants	0.0493 ***	0.0449 ***	0.0535 ***	0.0847 ***	0.0565 ***	0.0793 ***
	(0.0150)	(0.0153)	(0.0124)	(0.0183)	(0.0179)	(0.0184)
logHealth_ Medical_productspc	0.363 ***					
	(0.0365)					
logHealth_ Outpatient_servicespc		0.308 *** (0.0272)				
logHealth_ Hospital_servicespc			0.613 *** (0.0386)			
logHealth_Public_health_servicespc				0.0700 *** (0.0180)		
logHealth_ R&D_Healthpc					0.0943 *** (0.0149)	
logHealth_ n.e.cp						0.0723 *** (0.0171)
PHealthInmig_VGood16_64	0.000488 (0.00119)	0.00536 *** (0.00134)	−0.000155 (0.00101)	0.00101 (0.00180)	0.00137 (0.00140)	0.00188 (0.00177)
Ageing	0.00986 (0.00990)	−0.0118 (0.0101)	−0.0261 *** (0.00897)	−0.00209 (0.0126)	0.00889 (0.0110)	0.00567 (0.0125)
Constant	7.369 *** (0.252)	7.845 *** (0.239)	5.893 *** (0.246)	8.866 *** (0.251)	9.017 *** (0.243)	8.723 *** (0.249)
Observations	333	333	333	326	310	333
R-squared	0.991	0.991	0.994	0.987	0.991	0.987
Number of Idcountry	27	27	27	27	26	27

Own elaboration by the authors using Stata. Standard errors in parentheses *** *p* < 0.01.

**Table 5:** Prais–Winsten Regression models with breakdown of public health expenditure by function and countries.

Models	Prais–Winsten Regression	Prais–Winsten Regression	Prais–Winsten Regression	Prais–Winsten Regression	Prais–Winsten Regression	Prais–Winsten Regression	Prais–Winsten Regression
	Model 15	Model 16	Model 17	Model 18	Model 19	Model 20	Model 21
Variables	logGDPpc	logGDPpc	logGDPpc	logGDPpc	logGDPpc	logGDPpc	logGDPpc
logImmigrants	0.0576 ***	0.0598 ***	0.0691 ***	0.0610 ***	0.0719 ***	0.0764 ***	0.0742 ***
	(0.0113)	(0.0129)	(0.0142)	(0.0116)	(0.0141)	(0.0150)	(0.0145)
logHealth_Medical_productspc	0.407 ***						
	(0.0419)						
PHealthInmig_VGood16_64	0.000279 (0.000769)	0.000556 (0.000739)	0.000869 (0.000846)	9.78 × 10^−5^ (0.000806)	−0.000148 (0.000811)	0.00164 * (0.000988)	0.000555 (0.000830)
ageing_65	0.0216 *** (0.00561)	0.0407 *** (0.00578)	0.0418 *** (0.00650)	0.0161 *** (0.00576)	0.0505 *** (0.00634)	0.0378 *** (0.00662)	0.0438 *** (0.00630)
logHealth_Medical_productspc		0.246 *** (0.0366)					
logHealth_Outpatient_servicespc			0.0518 ** (0.0227)				
logHealth_Hospital_servicespc				0.365 *** (0.0412)			
logHealth_Public_health_servicespc					0.00182 (0.00862)		
logHealth_R&D_Healthpc						−0.0111 (0.0111)	
logHealth_n.e.cpc							0.0253 ** (0.0127)
2.wave (2013–2017)	0.0215 ** (0.0109)	0.0114 (0.0113)	0.00930 (0.0122)	0.0275 ** (0.0112)	0.0136 (0.0118)	0.0195 (0.0130)	0.00932 (0.0124)
3.wave (2018–2020)	0.0391 ** (0.0169)	0.0416 ** (0.0174)	0.0555 *** (0.0181)	0.0562 *** (0.0167)	0.0605 *** (0.0174)	0.0716 *** (0.0193)	0.0564 *** (0.0185)
Belgium	−0.0437 *** (0.0159)	0.0124 (0.0218)	−0.109 *** (0.0222)	0.00235 (0.0207)	−0.0781 *** (0.0185)	−0.120 *** (0.0423)	−0.0643 *** (0.0199)
Denmark	0.128 *** (0.0191)	0.327 *** (0.0188)	0.243 *** (0.0182)	0.0553 ** (0.0265)	0.244 *** (0.0196)	0.238 *** (0.0190)	0.238 *** (0.0191)
France	−0.178 *** (0.0240)	−0.265 *** (0.0264)	−0.284 *** (0.0280)	−0.0930 *** (0.0304)	−0.271 *** (0.0290)	−0.279 *** (0.0307)	−0.233 *** (0.0336)
Germany	−0.172 *** (0.0341)	−0.380 *** (0.0320)	−0.345 *** (0.0372)	−0.0360 (0.0465)	−0.367 *** (0.0394)	−0.349 *** (0.0432)	−0.362 *** (0.0382)
Italy	−0.286 *** (0.0386)	−0.427 *** (0.0350)	−0.560 *** (0.0330)	−0.200 *** (0.0491)	−0.595 *** (0.0323)	−0.557 *** (0.0390)	−0.535 *** (0.0369)
Luxembourg	0.861 *** (0.0399)	0.786 *** (0.0605)	1.099 *** (0.0416)	0.981 *** (0.0338)	1.159 *** (0.0364)	1.118 *** (0.0386)	1.152 *** (0.0375)
Netherlands	0.0534 *** (0.0196)	0.110 *** (0.0211)	0.0425 (0.0258)	0.120 *** (0.0225)	0.0683 *** (0.0258)	0.0662 *** (0.0218)	0.0690 *** (0.0235)
Sweden	0.101 *** (0.0191)	0.132 *** (0.0277)	0.0180 (0.0368)	0.256 *** (0.0297)	0.0539 (0.0335)	0.0535 (0.0328)	0.0707 ** (0.0331)
Finland	0.0751 *** (0.0224)	0.146 *** (0.0299)	−0.0155 (0.0337)	0.184 *** (0.0265)	0.0169 (0.0370)	0.0275 (0.0319)	0.0554 * (0.0313)
Greece	−0.390 *** (0.0649)	−0.808 *** (0.0502)	−0.795 *** (0.0817)	−0.446 *** (0.0641)	−1.043 *** (0.0600)	−0.951 *** (0.0775)	−0.823 *** (0.0744)
Ireland	0.379 *** (0.0676)	0.512 *** (0.0721)	0.443 *** (0.0822)	0.519 *** (0.0662)	0.551 *** (0.0745)	0.409 *** (0.0843)	0.489 *** (0.0753)
Malta	−0.0856 * (0.0467)	−0.174 *** (0.0553)	−0.405 *** (0.0470)	−0.174 *** (0.0414)	−0.440 *** (0.0424)	−0.476 *** (0.0815)	−0.424 *** (0.0457)
Portugal	−0.365 *** (0.0472)	−0.400 *** (0.0609)	−0.750 *** (0.0363)	−0.388 *** (0.0483)	−0.796 *** (0.0432)	−0.763 *** (0.0400)	−0.740 *** (0.0387)
Spain	−0.285 *** (0.0438)	−0.456 *** (0.0376)	−0.595 *** (0.0333)	−0.200 *** (0.0561)	−0.606 *** (0.0350)	−0.615 *** (0.0333)	−0.545 *** (0.0451)
Cyprus	0.255 *** (0.0739)	−0.0203 (0.0590)	−0.126 * (0.0728)	0.104 * (0.0601)	−0.205 *** (0.0595)	−0.330 *** (0.0671)	−0.141 * (0.0740)
Bulgaria	−0.793 *** (0.0982)	−1.172 *** (0.0832)	−1.578 *** (0.0759)	−0.933 *** (0.0938)	−1.731 *** (0.0513)		−1.662 *** (0.0506)
Czechia	−0.426 *** (0.0392)	−0.526 *** (0.0414)	−0.721 *** (0.0318)	−0.385 *** (0.0465)	−0.761 *** (0.0308)	−0.786 *** (0.0405)	−0.729 *** (0.0310)
Slovakia	−0.264 *** (0.0537)	−0.463 *** (0.0522)	−0.586 *** (0.0497)	−0.251 *** (0.0563)	−0.595 *** (0.0499)	−0.661 *** (0.0716)	−0.588 *** (0.0505)
Estonia	−0.307 *** (0.0640)	−0.499 *** (0.0649)	−0.699 *** (0.0774)	−0.452 *** (0.0575)	−0.823 *** (0.0674)	−0.787 *** (0.0692)	−0.740 *** (0.0696)
Latvia	−0.282 *** (0.0838)	−0.574 *** (0.0785)	−0.927 *** (0.0748)	−0.355 *** (0.0845)	−1.035 *** (0.0700)	−1.116 *** (0.114)	−0.962 *** (0.0710)
Hungary	−0.479 *** (0.0718)	−0.842 *** (0.0528)	−1.076 *** (0.0412)	−0.440 *** (0.0847)	−1.144 *** (0.0319)	−1.176 *** (0.0530)	−1.106 *** (0.0345)
Lithuania	−0.379 *** (0.0730)	−0.671 *** (0.0652)	−0.952 *** (0.0614)	−0.368 *** (0.0802)	−1.030 *** (0.0588)	−1.030 *** (0.0779)	−0.976 *** (0.0574)
Croatia	−0.554 *** (0.0640)	−0.955 *** (0.0438)	−1.057 *** (0.0540)	−0.547 *** (0.0695)	−1.152 *** (0.0421)	−1.170 *** (0.0680)	−1.116 *** (0.0406)
Slovenia	−0.247 *** (0.0396)	−0.391 *** (0.0356)	−0.549 *** (0.0322)	−0.214 *** (0.0459)	−0.577 *** (0.0316)	−0.581 *** (0.0432)	−0.546 *** (0.0327)
Poland	−0.518 *** (0.0775)	−0.142 (0.163)	−1.112 *** (0.0571)	−0.646 *** (0.0721)	−1.158 *** (0.0537)	−1.220 *** (0.0578)	−1.120 *** (0.0591)
Romania	−0.718 *** (0.103)	−1.263 *** (0.0678)	−1.449 *** (0.104)	−0.802 *** (0.110)	−1.639 *** (0.0498)	−1.782 *** (0.0959)	−1.638 *** (0.0422)
Constant	6.216 *** (0.301)	7.618 *** (0.242)	8.648 *** (0.203)	6.826 *** (0.267)	8.809 *** (0.176)	8.992 *** (0.201)	8.770 *** (0.190)
Observations	333	333	333	333	326	310	333
R-squared	0.996	0.997	0.997	0.996	0.998	0.997	0.997
Number of Idcountry	27	27	27	27	27	26	27

Own elaboration by the authors using Stata. Base wave: 1 wave (2008–2012); base country: Austria. Standard errors in parentheses *** *p* < 0.01, ** *p* < 0.05, * *p* < 0.1.

**Table 6:** Prais–Winsten Regression models with different variables representing immigration without Ageing_65 variable.

Models	Prais–Winsten Regression	Prais–Winsten Regression	Prais–Winsten Regression	Prais–Winsten Regression
	Model 22	Model 23	Model 24	Model 25
Variables	logGDPpc	logGDPpc	logGDPpc	logGDPpc
logImmigrants	0.00427			
	(0.00890)			
logImmigrantswom		0.00375		
		(0.00889)		
logImmigrantsmen			0.00375	
			(0.00911)	
logImmigyears15_64				0.00509
				(0.0125)
logHealthpc	0.724 ***	0.726 ***	0.722 ***	0.831 ***
	(0.0211)	(0.0213)	(0.0210)	(0.0264)
PHealthImmig_VGood16_64	0.00173 **	0.00180 **	0.00168 *	0.000317
	(0.000871)	(0.000874)	(0.000868)	(0.000859)
Ageing_65				
Constant	4.691 ***	4.683 ***	4.709 ***	3.954 ***
	(0.150)	(0.144)	(0.153)	(0.185)
Observations	333	333	333	179
R-squared	0.996	0.996	0.996	0.998
Number of Idcountry	27	27	27	20

Own elaboration by the authors using Stata. Standard errors in parentheses *** *p* < 0.01, ** *p* < 0.05, * *p* < 0.1.

**Table 7:** Means of country coefficients of Prais–Winsten models Regression models with breakdown of public expenditure on health by function.

Models Variables	Model 15	Model 16	Model 17	Model 18	Model 19	Model 20	Model 21	Mean
	logGDPpc	logGDPpc	logGDPpc	logGDPpc	logGDPpc	logGDPpc	logGDPpc	
Romania	−0.7180	−1.2630	−1.4490	−0.8020	−1.6390	−1.7820	−1.6380	−1.3273
Bulgaria	−0.7930	−1.1720	−1.5780	−0.9330	−1.7310		−1.6620	−1.1241
Croatia	−0.5540	−0.9550	−1.0570	−0.5470	−1.1520	−1.1700	−1.1160	−0.9359
Hungary	−0.4790	−0.8420	−1.0760	−0.4400	−1.1440	−1.1760	−1.1060	−0.8947
Poland	−0.5180	−0.1420	−1.1120	−0.6460	−1.1580	−1.2200	−1.1200	−0.8451
Lithuania	−0.3790	−0.6710	−0.9520	−0.3680	−1.0300	−1.0300	−0.9760	−0.7723
Greece	−0.3900	−0.8080	−0.7950	−0.4460	−1.0430	−0.9510	−0.8230	−0.7509
Latvia	−0.2820	−0.5740	−0.9270	−0.3550	−1.0350	−1.1160	−0.9620	−0.7501
Czechia	−0.4260	−0.5260	−0.7210	−0.3850	−0.7610	−0.7860	−0.7290	−0.6191
Estonia	−0.3070	−0.4990	−0.6990	−0.4520	−0.8230	−0.7870	−0.7400	−0.6153
Portugal	−0.3650	−0.4000	−0.7500	−0.3880	−0.7960	−0.7630	−0.7400	−0.6003
Slovakia	−0.2640	−0.4630	−0.5860	−0.2510	−0.5950	−0.6610	−0.5880	−0.4869
Spain	−0.2850	−0.4560	−0.5950	−0.2000	−0.6060	−0.6150	−0.5450	−0.4717
Italy	−0.2860	−0.4270	−0.5600	−0.2000	−0.5950	−0.5570	−0.5350	−0.4514
Slovenia	−0.2470	−0.3910	−0.5490	−0.2140	−0.5770	−0.5810	−0.5460	−0.4436
Malta	−0.0856	−0.1740	−0.4050	−0.1740	−0.4400	−0.4760	−0.4240	−0.3112
Germany	−0.1720	−0.3800	−0.3450	−0.0360	−0.3670	−0.3490	−0.3620	−0.2873
France	−0.1780	−0.2650	−0.2840	−0.0930	−0.2710	−0.2790	−0.2330	−0.2290
Belgium	−0.0437	0.0124	−0.1090	0.0024	−0.0781	−0.1200	−0.0643	−0.0572
Cyprus	0.2550	−0.0203	−0.1260	0.1040	−0.2050	−0.3300	−0.1410	−0.0662
Finland	0.0751	0.1460	−0.0155	0.1840	0.0169	0.0275	0.0554	0.0699
Netherlands	0.0534	0.1100	0.0425	0.1200	0.0683	0.0662	0.0690	0.0756
Sweden	0.1010	0.1320	0.0180	0.2560	0.0539	0.0535	0.0707	0.0979
Denmark	0.1280	0.3270	0.2430	0.0553	0.2440	0.2380	0.2380	0.2105
Ireland	0.3790	0.5120	0.4430	0.5190	0.5510	0.4090	0.4890	0.4717
Luxembourg	0.8610	0.7860	1.0990	0.9810	1.1590	1.1180	1.1520	1.0223
Austria (base country)	0	0	0	0	0	0	0	0
Constant	6.216 ***	7.618 ***	8.648 ***	6.826 ***	8.809 ***	8.992 ***	8.770 ***	

Own elaboration by the authors. Standard errors in parentheses *** *p* < 0.01.

## Data Availability

Data from The World Bank and Eurostat databases have been used: https://databank.worldbank.org/home.aspx (accessed on 11 October 2022); https://ec.europa.eu/eurostat/data/database (accessed on 13 October 2022).
